# Protocol for a virtual nominal group technique to develop expert consensus on graded return to sports, exercise and physical activity during intermediate and late-phase rehabilitation following spinal fusion in AIS

**DOI:** 10.1136/bmjopen-2025-107478

**Published:** 2025-11-16

**Authors:** Susanna Tucker, Nicola R Heneghan, Adrian Gardner, Alison B Rushton, Emily Russell, Andrew Soundy

**Affiliations:** 1 School of Sport, Exercise and Rehabilitation Sciences, University of Birmingham - Edgbaston Campus, Birmingham, England, UK; 2 Royal Orthopaedic Hospital NHS Foundation Trust, Birmingham, England, UK; 3 School of Sport, Exercise and Rehabilitation Sciences, University of Birmingham, Birmingham, England, UK; 4 Institute of Health and Neurodevelopment, Aston University, Birmingham, England, UK; 5 Spinal Services, Royal Orthopaedic Hospital, Birmingham, England, UK; 6 School of Physical Therapy, Western University Faculty of Health Sciences, London, England, Canada; 7 Buckinghamshire Healthcare NHS Trust, Amersham, England, UK

**Keywords:** Scoliosis, PAEDIATRICS, ORTHOPAEDIC & TRAUMA SURGERY, Spine, REHABILITATION MEDICINE

## Abstract

**Abstract:**

**Introduction:**

Adolescent idiopathic scoliosis (AIS) is a common paediatric spinal deformity with large curves surgically managed through spinal fusion. However, postoperative rehabilitation remains inconsistent and varies depending on clinician, hospital or location. Our international e-Delphi consensus established a broad range of statements from preoperative care until 12 months postoperatively. However, rehabilitation and graded return to sport between 3 and 12 months remains vague and further consensus work is needed. This study aims to understand the intermediate and late stages of rehabilitation in order to guide return to sport, exercise and physical activity. The primary objective is to explore content of rehabilitation and milestones between 3 and 12 months postoperatively. This understanding of postoperative care will form the basis for future postoperative guidance.

**Methods and analysis:**

This protocol for a nominal group technique (NGT) study is written in accordance with the Accurate Consensus Reporting Document guidelines. A national sample of expert surgeons, physiotherapists and nurses in AIS will be recruited. The NGT will take place virtually and will consist of six stages: stage 1: idea generation; stage 2: round robin idea sharing; stage 3: discussion and clarification; stage 4: anonymous voting; stage 5: results feedback; and stage 6: discussion and final voting. This NGT will be preceded by a scoping review which will be disseminated a priori to inform stage 1 idea generation. The population, concept, context framework will be used to explore postoperative rehabilitation towards sports, exercise or physical activities following any kind of spinal surgery. The study steering group and patient and public involvement representative have been involved from conceptualisation and will continue to be involved until final dissemination.

**Ethics and dissemination:**

The University of Birmingham has provided ethical approval: ERN_4201-Jun2025. Dissemination will take place through conference presentation and peer-reviewed publications.

**STRENGTHS AND LIMITATIONS**

- Participant heterogeneity regarding profession (surgeons, nurses or physiotherapists) but homogeneity regarding expertise and area of interest (AIS) facilitates both critical debate and greater exploration of ideas.
- Nominal group technique (NGT) facilitates structured and in-depth exploration of ideas and discussion towards consensus where our previous Delphi was unable to do so.
- Face-to-face NGT helps promote richness of discussion and critical evaluation of ideas.
- Combining this NGT with previous e-Delphi consensus helps improve generalisability of consensus.
- Geographical location and time required to attend a face-to-face NGT may preclude some individuals from participating.

## Introduction

 Adolescent idiopathic scoliosis (AIS) is a three-dimensional deformity of the spine and one of the most common paediatric spinal deformities present in approximately 2%–3% of the general population.^1^,^5^ Curves that exceed 50 degrees are often surgically managed through spinal fusion.^3 6^ Postoperatively, 28%–37% of individuals choose lower impact activities due to a loss of flexibility, pain, fear and deconditioning.^7^ Postoperative functional deficits include reduced lung function, joint range of motion and reduced strength, thereby predisposing individuals to sport-related injury if not appropriately addressed during rehabilitation.^8^ Complications from participation in sport following scoliosis fusion surgery, such as neurological injury, anchor failures, loosening of implants, rod failure, pseudoarthrosis and loss of correction, are rarely reported.^9^ Sports, exercise and physical activity are identified as effective in reducing the need for further operative intervention and level of disability, improving satisfaction with function as well as having socioeconomic benefits for the individual.^10^

Several factors may limit postoperative return to sports. These may include functional factors such as the inferior limit to the fusion.^11 12^ Additionally, social factors including behavioural development during adolescence and parental influence may also be responsible for decreased activity levels.^7^ The attitude and support of healthcare professionals are key drivers in determining whether an individual will return to sports.^9 13^ Furthermore, the delivery of postoperative rehabilitation for AIS is reported as globally inconsistent and varied based on clinician, hospital or location.^14^ Therefore, there is an urgent need for the development of a consensus statement that can inform future guidance and facilitate consistent care regardless of clinician or location.^13^ Our international e-Delphi study provided the first postoperative consensus on return to sport, exercise and physical activity following spinal fusion in AIS.^15^ A broad range of consensus statements were developed from preoperative care until 12 months postoperatively with agreement among experts that return to sport, exercise and physical activity needs to be graded.^15^ Early stages of rehabilitation until 3 months postoperatively were well defined and detailed; however, graded return from 3 to 12 months remained vague.^15^ Further consensus is needed to understand the agreed needs from acute to longer-term rehabilitation, ensuring that rehabilitation is appropriately graded and progressive.^15^

Nominal group technique (NGT) was chosen to follow our previous e-Delphi study to help identify the intervention in accordance with the updated Medical Research Council (MRC) framework for complex interventions.^15 16^ This framework has been used due to its ability to ask a broader range of questions regarding the impact, value, resources, and theorising how the intervention works in relation to its context and contributes to system change and real world decision making.^16^ Furthermore, the choice to complete an NGT to determine a firm understanding of postoperative care in AIS from 3 months onwards allows us to answer broader complex rehabilitation questions that are useful to clinicians in a way that the Delphi was unable to do so.^16^ Ensuring understanding of rehabilitation via NGT is necessary and superior over automatic progression to the next phase of the MRC framework when uncertainties remain unresolved.^16^ This NGT will identify aspects of the Delphi consensus needing further work for application with consideration of the core elements: context, programme theory, stakeholders, uncertainties, refinement and economic considerations.^16^ Furthermore, by using evidence alongside the formulation of consensus with affected healthcare professionals, it is hoped that this NGT consensus alongside the Delphi consensus will be able to formulate guidance that can then be implemented in clinical practice.^17^

### Aims and objectives

This study aims to understand the intermediate and late stages of rehabilitation to guide return to sport, exercise and physical activity following fusion surgery for AIS. This understanding of postoperative care will form the basis for future post-op guidance.

The first objective of this study is to complete an NGT with UK-based physiotherapists, surgeons and nurses who have experience in rehabilitation following spinal fusion for AIS. Second, to explore content, milestones and graded return to sports following spinal fusion in AIS during the intermediate and late stages of rehabilitation between 3 and 12 months postoperatively. Finally, to saturate and secure a firm understanding of postoperative care and thereby form the basis for a future guidance document.

## Methods

This study will combine NGT with our previous e-Delphi results to explore ideas in relation to the later stages of rehabilitation for return to sports, exercise and physical activity.^18^ The NGT is a highly structured discussion which empowers participants, allowing their voices to be heard and opinions considered, typically resulting in higher concordance between participants, thereby increasing agreement and enabling creation of consensus statements due to the exploration of discordant ideas where a Delphi was unable to do so due to the absence of discussion.^18 19^ This hybrid approach, combining Delphi methods with NGT to form a consensus, allows the reliability of a Delphi with an increased number of participants and equitable levelling of contributions; meanwhile, the NGT meeting allows better understanding and results, exploration of viewpoints, and prompt results to inform future postoperative guidance.^18 19^

This study will be reported in accordance with the Accurate Consensus Reporting Document (ACCORD) guidelines and subheadings with corresponding number references to the checklist.^20^ The ACCORD guideline is recommended by the Enhancing the QUAlity and Transparency Of health Research (EQUATOR) network and is designed to improve transparency and completeness of reporting, improving rigour.^20 21^

A pragmatic philosophical stance is adopted, which allows the researcher to use multiple sources of data and knowledge to investigate patient-orientated issues.^22^ Furthermore, pragmatism encourages a focus on different possible solutions to the lack of guidance in postoperative rehabilitation and allows either acceptance or rejection of ideas democratically.^22^ Pragmatism has previously been successfully used in NGT consensus generation and was therefore chosen for this project.^23^

### M1 Registration

Due to an absence of appropriate registries for consensus protocols, this protocol has instead been published in order to obtain external peer review on methods, proof of priority of ideas and increase visibility to the research community.^24^

### M2 Participants

Physiotherapists, surgeons and nurses were chosen as the population of interest for the NGT as the consensus generated will be designed to inform future clinical guidance, improve consistency of rehabilitation and assure clinicians about the appropriateness of their treatment.^13^ The heterogeneity of the group is advantageous when developing consensus due to consideration of different perspectives, alternative treatments, exploration of uncertainty, and creative and strategic decision making.^25^ In contrast, some evidence suggests that group homogeneity reduces power differentials, allowing participants to freely express their views and potentially contradict views of other participants in discussion towards consensus.^18^ However, the benefits of NGT heterogeneity outweigh that of homogeneity, with the rigour and structure of NGT facilitating consensus among relevant professionals with different perspectives.^25^,^27^ Therefore, this NGT will allow heterogeneity with regard to attributes such as profession or location and homogeneity with regard to area of expertise (AIS) so that conflict can be constructively managed that might otherwise occur in a heterogenous group without shared expertise.^25 27^ Any conflict during the NGT resulting from heterogeneous professions will be managed during open discussions with critical evaluation to ultimately steer the group towards a consensus that overcomes controversy.^25^

### M3 Panel numbers

NGT has been documented with between 4 and 121 participants.^28 29^ This NGT will aim to recruit up to 14 participants distributed across expert surgeons, nurses and physiotherapists.^18^

In order to maintain group homogeneity with regard to expertise and intercorrelation, participants were eligible if they were deemed an expert within AIS.^25 30^ For the purpose of the NGT, an expert was considered as having sufficient knowledge regarding the topic of interest.^31^ Therefore, it was decided that individuals must meet at least one of the following criteria in keeping with our previous Delphi study expert definition.^15 32^

1. ≥2 publications on AIS, paediatric spinal pain or adolescent spinal pain in peer-reviewed journals in the last 10 years.^32^
2. A caseload of ≥20% spinal deformity practice specifically in AIS (either conservative or operative) per month. No exclusion criteria will be applied with regard to geographical location or cultural background.^32^

Participant selection will be led by (ST) in discussion with the study steering group (AS, NH, AG, AR) and completed on a first come first serve basis provided individuals meet the expert criteria.

### M4 Participant recruitment

Recruitment of surgeons, physiotherapists, and nurses will take place through professional networks, charities, social media snowballing and literature searches.^15 25 33 34^

### M5 Patient and Public Involvement

Prior to completion of the NGT, a Patient and Public Involvement and Engagement (PPIE) group was created using 3–4 patients (identified as experts having undergone spinal fusion for AIS). PPIE discussions and results will be reviewed by a methodologist (AS), physiotherapy subject experts (ST, AR, NH) and a surgeon (AG). PPIE expert patients will be invited to discuss their experiences of rehabilitation and help shape questions used during NGT. PPIE is a core element within the MRC framework and needs to be considered early and continually revised at each stage of the research process facilitating concordance between patient preference and clinical guidance regarding treatment.^16 35^

### M6 Preparatory research

In order to develop the consensus, the identification of how preparatory research was obtained is required. For the purpose of this research, items were initially generated through three stages. Stage 1: items will be generated initially by the results of our past Delphi study. Stage 2: items will be generated and supplemented by a scoping review. Stage 3: items will be integrated and generated from stages 1 and 2.

Stage 1: previous e-Delphi results will be shared including the published article that is publicly available, a list of consensus items that were generated, and a visualisation of consensus items in the form of a figure that summarises results.^15^

Stage 2: a scoping review of research will be completed using the population, concept, context (PCC) framework as outlined below.^36^

Stage 3: a list of articles as well as a tabular summary of the literature with a data charting form completed for each article (online supplemental Appendix 3) will be presented to participants a priori and is described below.

### M7 Literature searching scoping review

The scoping review will be completed prior to the NGT using a six-stage approach (online supplemental Appendix 4) shown to both increase reliability and rigour.^37^ This six-stage approach has been shown to aid in producing in-depth and broad results identifying all relevant literature regardless of study design.^37^ In addition, this framework will also draw on the PCC framework outlined by the Joanna Briggs Institute due to its broader scope and less restrictive inclusion criteria.^36 38 39^ Additional enhancements to clarify each stage of the literature searching have more recently been proposed to support the application and relevance of scoping studies and these will be included within our search.^39 40^ The sixth and final stage, consultation with stakeholders, is deemed as optional but will take place through both the PPI group and NGT with clinicians.^39 40^ The scoping review will be completed by the primary researcher (ST) and checked by a secondary researcher (AS) and then reported according to the Preferred Reporting Items for Systematic reviews and Meta-Analyses (PRISMA) Extension for Scoping Reviews (PRISMA-ScR) checklist (online supplemental Appendix 5).^41^ Comprehensive detailing of the search strategy including databases or grey literature will be documented using the Peer Review of Electronic Search Strategies (PRESS) guideline.^42^ A data charting or data extraction form will be available based on previous recommendations in order to align extracted data with research question and objectives.^43^

### M8 Preliminary literature provided a-priori

The objective of this stage is to provide information that aids participants during the NGT. Ideas obtained from previous literature reviews or surveys have been shown to achieve greater consultation thereby helping to saturate consensus items.^18^ Asynchronous formatting with information a priori such as email threads has been shown to be beneficial in giving participants time to prepare, overcoming some of the drawbacks of NGT such as insufficient reflection time.^29 44^

Information explaining process and background information will be provided to participants via email to provide a brief summary of the process and introduce any existing literature that may assist with idea generation during stage 1.^29^

Included in the email will be:

1. The data charting sheet summarising studies identified during the scoping review (online supplemental Appendix 3).
2. A PRISMA flow diagram to demonstrate the number of articles searched for during the scoping review (online supplemental Appendix 2).
3. A summary of our previous e-Delphi consensus including the published article that is publicly available, a list of consensus items that were generated, and a visualisation of consensus items in the form of a figure that summarises results.
4. A brief summary of the NGT process to remind participants of what to expect.
5. Two questions to aid stage 1 idea generation.

- What time or function-based milestones are important during late-stage rehabilitation?
- What should rehabilitation and a graded return to sports consist of during intermediate and late stages between 3 and 12 months postoperatively?

### M9-M15 Consensus methods

A virtual NGT will be used to gather panellist input. Online platforms have the benefits of recording and transcription as well as visualisation of ideas, for example, virtual whiteboards.^29^ Virtual NGT has favourable levels of data richness compared with face-to-face interactions, includes geographically dispersed participants, reduces time and expenses for travel and meetings, and enables flexibility in scheduling.^29 31 45^ Documented concerns with virtual NGT include accessibility and technical support, although post-pandemic, these virtual environments are now commonplace among National Health Service employees and academics with technical issues mostly resolved.^29 31 45^ For the purposes of this study, Microsoft Teams was chosen as the online conferencing tool of choice, being an established and freely accessible platform, no additional expertise required, and previously successfully documented in a variety of virtual NGTs.^29 31^

This NGT will attempt to form a consensus that sits alongside our e-Delphi results and ultimately aids clarity in practice.^13 44^ The virtual NGT will consist of six distinct stages that will take place during one virtual meeting over approximately 1–2 hours.^18 29^

#### Stage 1: idea generation

Objective: Silent generation of ideas to ensure that group conformity is avoided.^44^

Participants will be given 20 min to silently generate their ideas.^18^ Participants will be asked to generate ideas for both intermediate and late stages of rehabilitation between 3 and 12 months postoperatively.^15^ Participants will not be directly prompted by facilitators during this stage but allowed to refer back to information provided a priori.

#### Stage 2: round robin idea sharing

Objective: round robin sharing of ideas with option for participants to send ideas to the moderator prior to the meeting if they wish.^29^ Round robin sharing will happen first for the intermediate stage of rehabilitation, then once complete, it will be repeated for late-stage rehabilitation.

Participants will be invited one by one to share ideas regarding different phases of rehabilitation while the transcribing facilitator adds these to the shared whiteboard.^29^ Participants will not be allowed to discuss items with each other at this stage but are allowed to think of new ideas during this phase and share new ideas during their turn.^18^ This stage will take as long as required until no new ideas are generated.^18^ Participants will not be prompted during this stage.

#### Stage 3: discussion and clarification

Objective: discussion between participants to clarify ideas shared and transcribing facilitator will update shared whiteboard ensuring clear points to be voted on.

In the case of a large number of ideas generated (>20 ideas per category), ideas will be grouped with agreement from all participants.^18^ Participants are able to alter their ideas as well as generate grouping themes with group discussion to ensure participant understanding, although participants do not have to agree with all ideas listed.^18^ The facilitator (ST) will not direct participants during this stage with regard to content of ideas but can help to coordinate the conversation and prevent individual participants from dominating the discussion.^18^

#### Stage 4: anonymous voting

Objective: participants rank their preferences from ideas generated for each phase of rehabilitation.

Participants will be given the opportunity to confidentially and anonymously vote on ideas generated using a ranking sheet. Participants will be asked to rank the entire list of ideas with the most important ranked 1, the next most important ranked 2, until all ideas are numbered.^46^

Definition of consensus: numbers will be summed for each item and if greater than 5 statements per category, then the top 5 items for each category with the lowest score will be defined as consensus statements.^18 46^

#### Break

10 min break for participants while results are summed and presented.

#### Stage 5: results feedback

Objective: summed ranking scores will be presented to participants for final discussion.^18^

#### Stage 6: discussion and final voting

Objective: participants may revise their ranking of all ideas in each category until no further changes occur.

A numerical level of consensus has not been well documented; therefore, during the final round, participants will be given the opportunity to revise their original ranking until no further changes are observed with the most important ideas.^18^ Individual scoring will be done using another ranking sheet and will remain confidential.^18^

Definition of consensus: score for each item will be summed and the top 5 lowest scoring items for each category will be included in the final consensus.^46^

### Data storage and handling

All information collected will be anonymised and grouped prior to final dissemination with individual demographics only visible to the research team. All participant data such as names, professional experience, location of work and email addresses will be securely stored using Research Electronic Data Capture (REDCap) on a password-protected computer with members of the research team only having access to the data. Following completion of the study, anonymised data will be securely stored within the University of Birmingham for 10 years following which it will be safely destroyed in accordance with the University of Birmingham guidelines.

Non-anonymised, confidential data such as signed consent forms and demographic data will also be collected and retained as part of the research process stored within REDCap. When the research project is completed and confidential personal data are no longer needed, then personal data will be deleted in accordance with the University’s High Level Data Retention Principles.

Data collected during the scoping review and NGT, including ideas and concepts discussed and transcribed during the meeting, will be collected and stored. There will be no audio or visual recording therefore eliminating any sensitive data. This data will be stored on password-protected university Microsoft 365 cloud storage.

Due to nature of the NGT, it is not possible to conceal participant identity including names, voices and image from other research participants during the data collection process. However, participants will be asked to retain confidentiality of other group members’ identity and content of NGT process.

### M16 pilot phase

The objective of the pilot phase is to ensure sufficient preparation of questions and formatting with a validity check in order to reduce risk of problems occurring during NGT.^47^ The preliminary literature and two questions will be presented to pilot participants followed by a pilot of the virtual NGT with a group of expert clinicians (surgeons, physiotherapists and nurses).

### M17 feedback to panellists

All participants will receive a copy of the final peer-reviewed journal article and further dissemination will take place through conference presentations.

### M18 Anonymity

Preliminary demographic data such as email addresses, years of experience and place of work will be visible only to the primary data collector (ST) and secondary reviewer (AS). This data will be anonymised and grouped into a participant demographics table prior to dissemination. However, due to the nature of virtual face-to-face meetings, it is not possible to conceal individual identity (names and image) from other members during data collection.

### M19 Study steering group

The study steering group was constituted of coauthors (AS, NRH, AG and AR) with methodological, academic and clinical expertise in physiotherapy and spinal surgery. The study steering group will meet a minimum of quarterly intervals to discuss the progress of the project from conception to final dissemination.

### M20 Incentives for participation

No financial incentives will be used for the purposes of this study.

### M21 Adaptations

Any adaptations made to the above protocol will be documented in the final manuscript.

## Discussion

### D2 Discussion of results and literature

This NGT has been designed to explore an existing gap in the literature regarding postoperative rehabilitation and return to sports, exercise and physical activity following spinal fusion in AIS. At present, postoperative rehabilitation following spinal fusion in AIS is highly variable depending on the healthcare provider.^14^ Previous literature has highlighted several disparities in management of AIS and the need for a multifaceted approach to improve equity in care.^48^ Furthermore, previous work has highlighted a lack of literature exploring exercise participation in AIS.^49^ Therefore, development of a consensus that can be used to inform future postoperative guidance and consistency of evidence-based care is warranted.^13^

This study aims to follow on from our previous e-Delphi which provided the first international expert consensus on postoperative rehabilitation with clearly defined statements regarding the first 3 months postoperatively but a lack of consensus regarding later stages of rehabilitation.^15^ A hybrid approach that combines a larger Delphi with a smaller NGT discussion improves reliability and broadens authority of results.^19^ Furthermore, NGT has been shown to have good application in healthcare settings in developing guidance or exploring opinions of healthcare professionals.^18 29^

Clinical guidance based on evidence, formulated by clinicians, with dissemination to relevant healthcare professionals for critique has the potential to improve equity of care within AIS.^17 48^ Furthermore, clinical guidance closes the gap between what clinicians do and what the evidence supports, improving consistency and quality of care.^13 35^ This NGT aims to understand the intermediate and late stages of postoperative rehabilitation by including current evidence, methodological expertise, stakeholders’ opinions and values, and clear standardised recommendations forming the basis for future post-op guidance.^50^ The decision to complete an NGT to increase understanding and generate consensus on intermediate and late-stage rehabilitation is consistent with previous literature which highlights that detailed and clear guidance and recommendations are twice as likely to be followed than vague non-specific recommendations.^51^ Results from the NGT and our previous e-Delphi results will form the basis document for future clinical guidance but require further testing in clinical practice including implementation fidelity and feedback for professionals based on their performance according to the guidance.^17^

## Supplementary material

10.1136/bmjopen-2025-107478 online supplemental file 1

10.1136/bmjopen-2025-107478 online supplemental file 2

10.1136/bmjopen-2025-107478 online supplemental file 3

10.1136/bmjopen-2025-107478 online supplemental file 4

## Data Availability

No data are available.
