# Supplementary material for: Protocol for a virtual nominal group technique to develop expert consensus on graded return to sports, exercise and physical activity during intermediate and late-phase rehabilitation following spinal fusion in AIS
Source: BMJ Open. 2025 Nov 16;15(11):e107478. doi: 10.1136/bmjopen-2025-107478 (PMC12625890; doi:10.1136/bmjopen-2025-107478)
Supplement: online supplemental file 1 [file bmjopen-15-11-s001.docx]

**Appendix 2:** PRISMA Flow Diagram

**Identification of studies via databases and registers**

Records removed *before screening*:

Duplicate records removed (n = )

Records marked as ineligible by automation tools (n = )

Records removed for other reasons (n = )

Records identified from*:

Databases (n =)

Registers (n = )

**Identification**

Records screened

(n = )

Records excluded**

(n = )

Reports sought for retrieval

(n = )

Reports not retrieved

(n = )

**Screening**

Reports assessed for eligibility

(n = )

Reports excluded:

Reason 1 (n = )

Reason 2 (n = )

Reason 3 (n = )

etc.

Studies included in review

(n = )

Reports of included studies

(n = )

**Included**
