# Supplementary material for: Protocol for a virtual nominal group technique to develop expert consensus on graded return to sports, exercise and physical activity during intermediate and late-phase rehabilitation following spinal fusion in AIS
Source: BMJ Open. 2025 Nov 16;15(11):e107478. doi: 10.1136/bmjopen-2025-107478 (PMC12625890; doi:10.1136/bmjopen-2025-107478)
Supplement: online supplemental file 2 [file bmjopen-15-11-s002.docx]

**Appendix 3: Data Charting Form**

1. **Bibliographic information**
   1. Study ID
   2. Article title
   3. Extracted by
   4. Checked by
   5. Type of publication (journal article, book chapter, grey literature)
   6. Country
2. **Researcher details**
   1. Authors and affiliations (list as presented on paper)
3. **Aims and methods**
4. Study aims/objectives
5. Methodology
6. Methods
7. **Scoping review PCC**
8. Population
9. Concept (interventions/programmes and outcomes assessed)
10. Context
11. **A priori themes (does the paper report data relating to the following?)**
12. What should graded return to sports, exercise, and physical activity during intermediate and late phase rehabilitation consist of?
13. What time or function-based milestones are important?
14. What should rehabilitation and a graded return to sports consist of during intermediate and late stages between 3 and 12 months post-operatively?
15. **Emergent themes (does the paper report on any further issues not related to the above that might be of interest to this review?)**
