# Supplementary material for: Protocol for a virtual nominal group technique to develop expert consensus on graded return to sports, exercise and physical activity during intermediate and late-phase rehabilitation following spinal fusion in AIS
Source: BMJ Open. 2025 Nov 16;15(11):e107478. doi: 10.1136/bmjopen-2025-107478 (PMC12625890; doi:10.1136/bmjopen-2025-107478)
Supplement: online supplemental file 3 [file bmjopen-15-11-s003.docx]

**Appendix 4:** Literature searching Framework

| **Stages** | **Arksey and O’Malley framework (**  **2005) [38].** | **Enhancements proposed by Levac, Colquhoun, and O’Brien (2010) [41]** | **Enhancements proposed by Peters et**  **al (2015) and Peters et**  **al (2020) [39, 40]** | **Implementation within this study** |
| --- | --- | --- | --- | --- |
| 1 | Identifying the research question | 1. Clearly articulate the research question that will guide the scope of inquiry. Consider the concept, target population, and health outcomes of interest to clarify the focus of the scoping study and establish an effective search strategy. | Title identifies the work as a scoping review with aspects of the PCC framework within the title. Objectives and questions clearly stated. The objective may be broad but can guide the scope of the enquiry. Review questions are consistent with title and thereby inform the inclusion criteria. | 1. What should graded return to sports, exercise, and physical activity during intermediate and late phase rehabilitation following spinal fusion in AIS consist of?  2. What time or function-based milestones are important during this stage?  3. What should rehabilitation and a graded return to sports consist of during intermediate and late stages between 3 and 12 months post-operatively? |
|  |  | 2. Mutually consider the purpose of the scoping study with the research question. Envision the intended outcome (e.g., framework, list of recommendations) to help determine the purpose of the study. |  | To explore content, milestones and graded return to sports during rehabilitation of other post-operative conditions during the intermediate and late stages of rehabilitation between 3 and 12 months.  To saturate and secure a firm understanding of post-operative care and rehabilitation in other conditions. |
|  |  | 3. Consider rationale for conducting the scoping study to help clarify the purpose. |  | To inform understanding of what concepts the literature suggests and to inform a discussion regarding what may be transferrable to this NGT and consensus work. |
| 2 | Identifying relevant studies | 1a. Research question and purpose should guide decision- making around the scope of the study. | Inclusion criteria will include types of sources or evidence used. Developing and aligning the inclusion  criteria with the objectives and  questions. Use of the population, concept, context framework with relevant details. | **Population:** Individuals undergoing any kind of surgical intervention, studies exploring post-operative care or rehabilitation following spinal surgery. Studies exploring conservative management or non-operative interventions will be excluded. No exclusion criteria will be applied with regard to type of surgery or participant demographics such as gender, age, ethnicity, or socioeconomic status.  **Concept:** Documents, evidences, experiences and perspectives that provide insight to participation or return to any kind of exercise, physical activity, sports, or rehabilitation following operative interventions.  **Context:** Studies must explore participation, uptake, engagement or involvement in sports, exercise, physical activity, or rehabilitation or literature exploring safety or ability of an individual to participate in sports, exercise or physical activity post-operatively.  **Other**: Articles where the full text is unavailable or that are not translated into English will be excluded. |
|  |  | 1b. Assemble a suitable team with content and methodological expertise that will ensure successful completion of the study. |  | The study steering group consisting of co-authors (AS, NRH, AG and AR) with methodological, academic and clinical expertise in physiotherapy and spinal surgery |
|  |  | 1c. When limiting scope is unavoidable, justify decisions and acknowledge the potential limitations to the study. |  | This scoping review will build upon previous work exploring sports, exercise, and physical activity participation in AIS which found an absence of literature. This scoping review is deliberately broad with a less restrictive inclusion criteria to aid development of an in-depth understanding of post-operative rehabilitation. |
| 3 | Study selection | 1. This stage will be considered an iterative process involving searching the literature, refining the search strategy, and reviewing articles for study inclusion. | Describing the planned approach to  evidence searching, selection. Comprehensive detailing of the search strategy including databases or grey literature. Use of the Peer Review of Electronic Search Strategies (PRESS) guideline [43]. Consideration of language of sources used. | Articles will be searched by two independent reviewers using Covidence software. The articles will first be screened by title followed by abstract. Any relevant studies will then be included for screening by full text. This process will be visualised using the PRISMA flow diagram (APPENDIX 2). PRESS guidelines will be used throughout [43].  **Information sources:** PubMed, EMBASE, MEDLINE, CINAHL Plus, Sport Discuss, SCOPUS, PubMed, PsycINFO, Nursing & Allied Health Database and Social Science Database. Grey literature will be searched using reference lists, Grey Matters, websites and search engines, for example, Google Scholar. |
|  |  | 2a. At the beginning of the process, the team will meet to discuss decisions surrounding study inclusion and exclusion. At least two reviewers will independently review abstracts for inclusion. |  | The study steering group (AS, NRH, AG and AR) will meet throughout the scoping review and NGT process to discuss all aspects of the study from conceptualisation until final dissemination. The primary researcher (ST) and secondary reviewer (AS) will review all abstracts for inclusion. |
|  |  | 2b. Reviewers will meet at the beginning, midpoint and final stages of the abstract review process to discuss challenges and uncertainties related to study selection and to go back and refine the search strategy if needed. |  | Reviewers (ST and AS) will meet at the point of screening titles, abstracts and full text for decisions and discuss any issues related to search strategy or study selection. |
|  |  | 2c. Two researchers will independently review full articles for inclusion. |  | Searches will be undertaken by the primary researcher (ST) and reviewed by the secondary researcher (AS) during July 2025 with specific dates listed. |
|  |  | 2d. When disagreements on study inclusion occur, a third reviewer can determine final inclusion. |  | Any disagreements on study inclusion will be discussed with an additional reviewer (NH) to determine final inclusion. |
| 4 | Charting the data | 1a. The research team should collectively develop the data- charting form and determine which variables to extract in order to answer the research question. | Searching for the evidence. Extracted data should align with research question and objectives. Data charting can also be known as data extraction. A charting table ought to be piloted and refined to communicate findings. Data extraction ought to involve at least two reviewers to reduce errors and bias. | The data charting form will be based on previous recommendations [44]. The data charting form will include bibliographic information, researcher details, aims and methods, scoping review PCC, A priori themes, emergent themes. The full data charting form is visible in (Appendix 3). |
|  |  | 1b. Charting should be considered an iterative process in which researchers continually extract data and update the data- charting form. |  | Data charting will be completing using the charting form, where necessary appropriate updates and amendments to the data charting form will be made and reviewed by the secondary reviewer (AS). |
|  |  | 1c. Two authors should independently extract data from the first five to ten included studies using the data-charting form and meet to determine whether their approach to data extraction is consistent with the research question and purpose. |  | Data charting will initially be completed by primary researcher (ST). The secondary researcher (AS) will also extract data from first ten studies to determine approach and consistent data extraction. |
|  |  | 2. Process-oriented data may require extra planning for analysis. A qualitative content analysis approach is suggested. |  | Where qualitative data is included then content analysis will be completed to ensure data is appropriately handled and summarised for NGT participants. |
| 5 | Collating, summarising and reporting the results | Researchers should break this stage into three distinct steps:  1a. Analysis (including descriptive numerical summary analysis and qualitative thematic analysis); | Selecting the evidence. Analysis ought to be predefined to improve transparency and justification of approach. Extracted data will then be used for a basic descriptive analysis which can be mapped into a visual presentation. | Themes identified from the data charting sheet will be summarised, tabulated with all corresponding citations visible for each theme. |
|  |  | 1b. Reporting the results and producing the outcome that refers to the overall purpose or research question; |  | Tabulated data summarising themes with citations, data charting sheets (Appendix 3), PRISMA flow diagram (Appendix 2), PRISMA Checklist (Appendix 4) and results will be presented to NGT participants for review a-priori. |
|  |  | 1c. Consider the meaning of the findings as they relate to the overall study purpose; discuss implications for future research, practice and policy. |  | Participants will be presented with summarised tabulated themes, original papers, and data charting sheets allowing individuals to opt for preferred levels of detail and information with preliminary literature a-priori. Participants will then be given two questions in advance to facilitate idea generation during stage 1 of NGT. |
| 6 | Consultation | 1. Consultation should be an essential component of scoping study methodology. | A visual presentation such as a diagrammatic, tabular, or descriptive form that aligns with the objectives and scope. Presentation in a suitable format allows identification of gaps in the literature and mapping of available evidence. | Consultation with clinicians will take place during NGT that is outlined below. |
|  |  | 2a. Clearly establish a purpose for the consultation. |  | The purpose of this scoping review is to then provide stakeholders with a broad in-depth summary of existing literature to aid generation of consensus on post-operative rehabilitation following spinal fusion in AIS. |
|  |  | 2b. Preliminary findings can be used as a foundation to inform the consultation. |  | Participants will be provided with results from scoping review to assist in idea generation during stage 1 of NGT. Participants will also be encouraged to generate any additional or broader ideas that may stem from clinical practice or further reading. |
|  |  | 2c. Clearly articulate the type of stakeholders to consult and how data will be collected, analysed, reported and integrated within the overall study outcome. |  | Consultation and data collection will follow NGT process clearly outlined below. The NGT will consist of six distinct stages to determine a consensus on post-operative rehabilitation. |
|  |  | 2d. Incorporate opportunities for knowledge transfer and exchange with stakeholders in the field. |  | The NGT includes opportunity for discussion and clarification of ideas based on idea generation that stems from existing literature predominantly identified during scoping review. |
