# Supplementary material for: Protocol for a virtual nominal group technique to develop expert consensus on graded return to sports, exercise and physical activity during intermediate and late-phase rehabilitation following spinal fusion in AIS
Source: BMJ Open. 2025 Nov 16;15(11):e107478. doi: 10.1136/bmjopen-2025-107478 (PMC12625890; doi:10.1136/bmjopen-2025-107478)
Supplement: Supplementary file 5 [file bmjopen-2025-107478.reviewer_comments.pdf]

PEER REVIEW HISTORY

BMJ Open publishes all reviews undertaken for accepted manuscripts. Reviewers are asked to complete a checklist review form and are provided with free text boxes to elaborate on their assessment. These free text comments are reproduced below.

ARTICLE DETAILS

Title (Provisional)

A protocol for a Virtual Nominal Group Technique to develop expert consensus on graded return to sports, exercise, and physical activity during intermediate and late phase rehabilitation following spinal fusion in AIS

Authors

Tucker, Susanna; Heneghan, Nicola R; Rushton, Alison B; Gardner, A.; Russell, E.; Soundy, Andrew

---

VERSION 1 - REVIEW

**Reviewer**	**1**
**Name**	**Kuru Çolak , Tuğba**
**Affiliation**	**Marmara University**
**Date**	**25-Sep-2025**
**COI**	**None**

Dear Editor,

Thank you for kindly inviting me to review the manuscript entitled “Nominal Group Technique to develop expert consensus on graded return to sports, exercise, and physical activity during intermediate and late phase rehabilitation following spinal fusion in AIS.”

I would like to congratulate the authors on their work. There is a significant gap in the literature in this area, and it is clear that the authors’ study will provide a valuable contribution.

My comments regarding the manuscript are as follows:

Abstract:

There is no need to use abbreviations for terms that appear only once in the abstract.

Please ensure that the keywords are written in accordance with MeSH terms.

Introduction:

There are some repetitions in this section. I suggest removing them and simplifying the text.

The “Objectives” section of the study may be written in plain text, in line with the journal’s guidelines.

Method:

The methods are described in sufficient detail and are appropriately structured.

General Comments:

In some parts of the manuscript, different characters have been used in the text. I recommend carefully revising the manuscript for consistency in writing.

I once again congratulate the authors on their valuable work.

VERSION 1 - AUTHOR RESPONSE

**Comment No.**	**Paragraph**	**Line No.**	**Reviewer / Editor Comment**	**Authors Action**
**Reviewer 1**				
1	Abstract	57<br>62-63	Abstract: There is no need to use abbreviations for terms that appear only once in the abstract.	Removal of ACCORD and PCC abbreviations as only appear once in abstract.
2	Abstract	63-64	Please ensure that the keywords are written in accordance with MeSH terms.	Key terms have been checked and against PubMed MESH term checker: https://www.ncbi.nlm.nih.gov/mesh/<br>Thank you.
3	Introduction	101<br>117	Introduction: There are some repetitions in this section. I suggest removing them and simplifying the text.	Removal of repetition: post-operatively in sentence.<br>Removal of repetition: This understanding of post-operative care will form the basis for future post op guidance.
4	Aims and Objectives	142-147	Introduction: The	Change of wording to continuous pros: ‘The first objective of this study is to complete a

			"Objectives" section of the study may be written in plain text, in line with the journal's guidelines.	NGT with UK based physiotherapists, surgeons, and nurses who have experience in rehabilitation following spinal fusion for AIS. Secondly, to explore content, milestones and graded return to sports following spinal fusion in AIS during the intermediate and late stages of rehabilitation between 3 and 12 months post-operatively. Finally, to saturate and secure a firm understanding of post-operative care and thereby form the basis for a future guidance document.'
5	Whole manuscript	1-475	General Comments: In some parts of the manuscript, different characters have been used in the text. I recommend carefully revising the manuscript for consistency in writing.	The manuscript has been revised and different characters amended to provide consistency in writing.
